# Restoration of regulatory and effector T cell balance and B cell homeostasis in systemic lupus erythematosus patients through vitamin D supplementation

**DOI:** 10.1186/ar4060

**Published:** 2012-10-17

**Authors:** Benjamin Terrier, Nicolas Derian, Yoland Schoindre, Wahiba Chaara, Guillaume Geri, Noël Zahr, Kubéraka Mariampillai, Michelle Rosenzwajg, Wassila Carpentier, Lucile Musset, Jean-Charles Piette, Adrien Six, David Klatzmann, David Saadoun, Cacoub Patrice, Nathalie Costedoat-Chalumeau

**Affiliations:** 1 UPMC Université Paris 6, UMR 7211, F-75013 Paris, France; 2 Centre National de la Recherche Scientifique (CNRS), UMR 7211, Paris, France; 3 INSERM, UMRS959; Groupe Hospitalier Pitié-Salpétrière, 47 boulevard de l'Hôpital, 75013 Paris, France; 4 Department of Internal Medicine, Hôpital Foch, 40 rue Worth, 92150 Suresnes, France; 5 Department of Biotherapy, Groupe Hospitalier Pitié-Salpétrière, 47 boulevard de l'Hôpital, 75013 Paris, France; 6 Department of Pharmacology, Groupe Hospitalier Pitié-Salpétrière, 47 boulevard de l'Hôpital, 75013 Paris, France; 7 Department of Internal Medicine, Groupe Hospitalier Pitié-Salpétrière, 47 boulevard de l'Hôpital, 75013 Paris, France; 8 P3S post-genomic platform, Groupe Hospitalier Pitié-Salpétrière, 47 boulevard de l'Hôpital, 75013 Paris, France; 9 Department of Immunology, Groupe Hospitalier Pitié-Salpétrière, 47 boulevard de l'Hôpital, 75013 Paris, France

## Abstract

**Introduction:**

Systemic lupus erythematosus (SLE) is a T and B cell-dependent autoimmune disease characterized by the appearance of autoantibodies, a global regulatory T cells (Tregs) depletion and an increase in Th17 cells. Recent studies have shown the multifaceted immunomodulatory effects of vitamin D, notably the expansion of Tregs and the decrease of Th1 and Th17 cells. A significant correlation between higher disease activity and lower serum 25-hydroxyvitamin D levels [25(OH)D] was also shown.

**Methods:**

In this prospective study, we evaluated the safety and the immunological effects of vitamin D supplementation (100 000 IU of cholecalciferol per week for 4 weeks, followed by 100 000 IU of cholecalciferol per month for 6 months.) in 20 SLE patients with hypovitaminosis D.

**Results:**

Serum 25(OH)D levels dramatically increased under vitamin D supplementation from 18.7±6.7 at day 0 to 51.4±14.1 (p<0.001) at 2 months and 41.5±10.1 ng/mL (p<0.001) at 6 months. Vitamin D was well tolerated and induced a preferential increase of naïve CD4^+ ^T cells, an increase of regulatory T cells and a decrease of effector Th1 and Th17 cells. Vitamin D also induced a decrease of memory B cells and anti-DNA antibodies. No modification of the prednisone dosage or initiation of new immunosuppressant agents was needed in all patients. We did not observe SLE flare during the 6 months follow-up period.

**Conclusions:**

This preliminary study suggests the beneficial role of vitamin D in SLE patients and needs to be confirmed in randomized controlled trials.

## Introduction

Systemic lupus erythematosus (SLE) is a systemic autoimmune disease characterized by skin, joint, neurological and kidney involvement and serositis. Therapeutic management depends on the type and severity of organ involvement and includes nonsteroidal anti-inflammatory drugs, hydroxychloroquine, corticosteroids, and immunosuppressive agents [1]. Nevertheless, long-term suppressive corticosteroids and/or immunosuppressive agents remain associated with morbidity and mortality [2]. SLE is a T and B cell-dependent disease characterized by the appearance of a variety of autoantibodies, some of which are pathogenic [1,3]. T cells are needed to initiate and sustain the secretion of antibodies by B cells, in particular to histones and double-stranded DNA [4]. SLE is also associated with global depletion of regulatory T cells (Tregs) [5], an increase in T helper lymphocytes producing IL-17 (Th17 cells) [6,7] and an increased expression of IFN-inducible genes [8].

Vitamin D from the skin and diet is metabolized in the liver to 25-hydroxyvitamin D (25(OH)D), which is used to determine a patient's vitamin D status; 25(OH)D is metabolized in the kidneys by the enzyme 25-hydroxyvitamin D-1α-hydroxylase (CYP27B1) to its active form, 1,25-dihydroxyvitamin D. Recent studies have shown the multifaceted immunomodulatory effects *in vitro *of active vitamin D (calcitriol or 1,25-dihydroxyvitamin D), which is the ligand of the vitamin D receptor, notably the expansion of Tregs, which are able to suppress proliferation of effector T cells [9], and the decrease of Th1 and Th17 cells [9,10]. Active vitamin D inhibits B cell activation and differentiation into plasmablasts and immunoglobulin production [10-12]. Active vitamin D has also been shown to inhibit the activation and maturation of dendritic cells [13]. In addition, studies have shown a significant correlation between higher SLE activity and lower serum 25(OH)D levels [13,14]. These findings provide a rationale for considering vitamin D supplementation as an immunomodulatory agent for SLE.

We report here on the findings of a preliminary prospective monocenter open-label study designed to assess the safety and immunological effects of oral vitamin D supplementation in patients with SLE. We showed that vitamin D supplementation modulates Tregs and effector T cell balance by increasing Tregs and decreasing the Th17 and Th1 cells, and it decreases memory B cells and anti-dsDNA levels.

## Materials and methods

### Patients

This prospective study included consecutive SLE patients from the Department of Internal Medicine at Pitié-Salpêtrière Hospital (http://www.clinicaltrials.gov NCT01413230). Patients were eligible for the study when they met at least four of the 1997 American College of Rheumatology criteria for SLE [15]. Inclusion criteria for the study were as follows: 1) inactive disease or mild to moderate active disease indicated by a score ≤ 8 in the Safety of Estrogens in Lupus Erythematosus National Assessment-Systemic Lupus Erythematosus Disease Activity Index (SELENA-SLEDAI), and 2) stable dosage of prednisone and/or immunosuppressive agents for at least 1 and/or 3 months, respectively. Pregnant patients and those planning pregnancy, and patients who had previously received B cell-targeted therapy were excluded. Disease activity was assessed using SELENA-SLEDAI [16].

### Study design

Between 1 September and 31 November 2010, we assessed 24 SLE patients for eligibility (twenty-two women and two men, mean age ± SD, 31 ± 8 years). Their serum 25(OH)D level was measured. Hypovitaminosis D was defined as serum 25(OH)D < 30 ng/mL, while vitamin D sufficiency was defined as serum levels between 30 and 100 ng/mL [17]. Those with hypovitaminosis D (< 30 ng/mL) were placed on the following schedule of oral vitamin D supplementation: 100,000 IU of cholecalciferol per week for 4 weeks, followed by 100,000 IU of cholecalciferol per month for 6 months. All supplemented patients were screened before vitamin D supplementation (Day 0, or D0), and 2 and 6 months (M2 and M6) after the beginning of vitamin D supplementation. All but four patients received hydroxychloroquine (200 or 400 mg daily) and/or oral prednisone (≤ 15 mg/day, median dosage 5 mg/day). Three patients received a stable dosage of immunosuppressive agents. The study was approved by the institutional ethics committee, the Comité de protection des personnes Ile-de-France VI, in the Pitié-Salpêtrière Hospital (Paris, France) and informed consent was obtained from all patients.

At each visit, the SELENA-SLEDAI was recorded. Routine measurements were made of 25(OH)D levels, antinuclear antibodies by indirect immunofluorescence on HEp2 cells (Immunoconcepts, Sacramento CA, USA), anti-dsDNA antibodies levels by ELISA (DiaSorin, Saluggia, Italy), complement C3 and C4 levels, complete blood count, serum creatinine, proteinuria and hematuria. Serum 25(OH)D was measured in serum samples by means of a radioimmunoassay after simple extraction with acetonitrile, as described previously [18].

The primary endpoint of this study was safety. Safety endpoints included the occurrence of hypercalcemia, hyperphosphoremia or lithiasis. Secondary outcomes included changes from baseline in T cell and B cell homeostasis, and cytokines and gene expression profiles, in peripheral blood mononuclear cells (PBMCs), and clinical efficacy assessment using the SELENA-SLEDAI.

### Flow cytometry

PBMC subsets (CD3^+^, CD4^+^, CD8^+ ^T lymphocytes, CD19^+ ^B lymphocytes and CD3^-^CD56^+ ^NK cells) counts (cells/μL) were established from fresh blood samples using CYTO-STAT tetraCHROME kits with Flowcount fluorescents beads as an internal standard and tetra CXP software with a Navios cytometer according to the manufacturer's instructions (Beckman Coulter, Villepinte, France). Subsets of these cells were analyzed using multicolor flow cytometry and monoclonal antibodies (mAbs) directly conjugated to various fluorescent markers. PBMCs were also stained with the following conjugated mAbs at predetermined optimal dilutions for 30 minutes at 4°C: CD3-ECD, CD4-PCy7, CD4-ECD, CD8-PCy7, CD8-APC, CD10-APC, CD16-FITC, CD19-ECD, CD27-PE, CD28-FITC, CD45RO-FITC, CD45RA-APC, CD56-PE, HLA-DR-PCy7 (Beckman Coulter), CD25-PE, CD38-PCy7, CD56-FITC, CD62L-FITC, IgD-FITC (BD Pharmingen, Le-Pont-de-Claix, France), CCR7-PE and LAP-PE (R&D systems, Lille, France), CD127-FITC (eBioscience, Paris, France) and GITR-PE and CD25-APC (Miltenyi Biotec, Paris, France). Intracellular detection of FoxP3 and CD152 (CTLA4) was performed on fixed and permeabilized cells using appropriate buffer (eBioscience for FoxP3, BD Pharmingen for CD152). Cell acquisition and analysis by flow cytometry were performed using a Navios Cytometer (Beckman Coulter). Instrument setting parameters (gains, compensations, and threshold) were set with machine software (CXP Software; Beckman Coulter) in conjunction with calibration beads (Flow-set beads, Cytocomp kit, and CYTO-TROL Control Cells). Machine reproducibility was verified with standardized beads (Flow-check). Data were analyzed with CXP analysis software and Kaluza software (Beckman Coulter)

For detection of intracellular cytokine production, PBMCs were stimulated with 50 ng/mL phorbol myristate acetate (PMA) and 1 mM ionomycin in the presence of Golgi-Plug (BD Pharmingen) for 4 hours and then stained with anti-IL-4-FITC, anti-IFN-γ-FITC, anti-IL-10-PE (BD Pharmingen), or anti-IL-17A-Alexa Fluor 647 (eBioscience) after fixation and permeabilization, according to the manufacturer's instructions. Culture supernatants from PBMCs stimulated in the absence of Golgi-Plug were harvested and immediately frozen at -80°C.

### Statistical analyses

We compared measures taken at baseline (before the vitamin D supplementation) with those taken at M2 and M6 after the initiation of vitamin D supplementation, using the Wilcoxon signed-rank test. All tests were two-sided with a 0.05 significance level. Graphing and statistical analyses were performed using Prism software (GraphPad Software, Inc.).

## Results

### Study design and participants

Out of the 24 patients screened for serum 25(OH)D levels, 20 patients (83%) had hypovitaminosis D and were included in this prospective study. The baseline characteristics of the 20 patients are listed in Table 1. All patients completed the 6 months follow-up period.

**Table 1:** Patient characteristics

Characteristics	N = 20
**Epidemiology**	
Age, y, mean ± SD	31 ± 8
Female gender, n (%)	20 (100)
**Previous SLE manifestations**	
Skin, n (%)	11 (55)
Joints, n (%)	9 (45)
Serositis, n (%)	4 (20)
Kidney, n (%)	11 (55)
Central nervous system, n (%)	1 (5)
SELENA-SLEDAI at Day 0, mean (range)	2 (0 to 8)
**25-Hydroxyvitamin D levels**	
Mean ± SD, ng/mL	22 ± 11
25(OH)D ≤ 10 ng/mL, n (%)	1 (5)
11 < 25(OH)D ≤ 20 ng/mL, n (%)	11 (55)
21 < 25(OH)D ≤ 30 ng/mL, n (%)	8 (40)
Associated treatments*	
Prednisone, n (%)	14 (70)
Prednisone, median, mg/day (range)	5 (0 to 15)
Hydroxychloroquine, n (%)	17 (85)
Azathioprine, n (%)	2 (10)
Mycophenolate mofetil, n (%)	1 (5)

*At the time of the study. SLE: systemic lupus erythematosus; SELENA-SLEDAI: Safety of Estrogens in Lupus Erythematosus National Assessment-Systemic Lupus Erythematosus Disease Activity Index; 25(OH)D: 25-hydroxyvitamin D.

### Safety and clinical and biological efficacy of vitamin D supplementation

**Figure 1:**
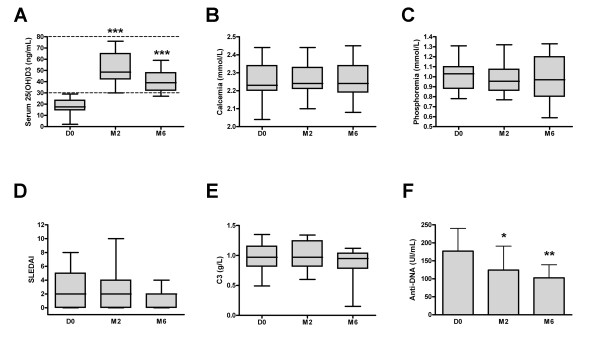
**Safety and clinico-biological efficacy of vitamin D supplementation in SLE patients**. Time evolution of serum 25(OH)D levels (**A**), disease activity assessed by the SLEDAI (**B**), and anti-dsDNA levels (**C**). Serum 25(OH)D levels dramatically increased with vitamin D supplementation, while anti-dsDNA levels decreased. The dotted line in panel A indicates normal values. Box plots indicate median, interquartile ranges, minimum and maximum values. Histograms indicate mean ± standard error of the mean (SEM). *P < 0.05, **P < 0.01, ***P < 0.001 versus Day 0; Wilcoxon test.

Serum 25(OH)D levels dramatically increased under vitamin D supplementation from 18.7 ± 6.7 at D0 to 51.4 ± 14.1 (*P *< 0.001) at M2 and to 41.5 ± 10.1 ng/mL (*P *< 0.001) at M6 (Figure 1A). Treatment was safe, with no significant increase of serum calcium and phosphorous and no occurrence of lithiasis. Although not statistically significant, disease activity assessed by the SELENA-SLEDAI slightly decreased from 2.9 ± 2.5 at D0 to 2.6 ± 2.5 at M2 (*P *= 0.67) and to 1.9 ± 1.8 at M6 (*P *= 0.16) (Figure 1B). C3 complement fraction levels remained stable during follow-up, while anti-dsDNA levels decreased from 177 ± 63 at D0 to 124 ± 67 at M2 (*P *< 0.05) and to 103 ± 36 IU/mL at M6 (*P *< 0.01) (Figures 1C). No patients required modification of the prednisone dosage or initiation of new immunosuppressant agents. We did not observe SLE flare during the 6 months follow-up period.

### Peripheral blood lymphocytes with vitamin D supplementation

**Figure 2:**
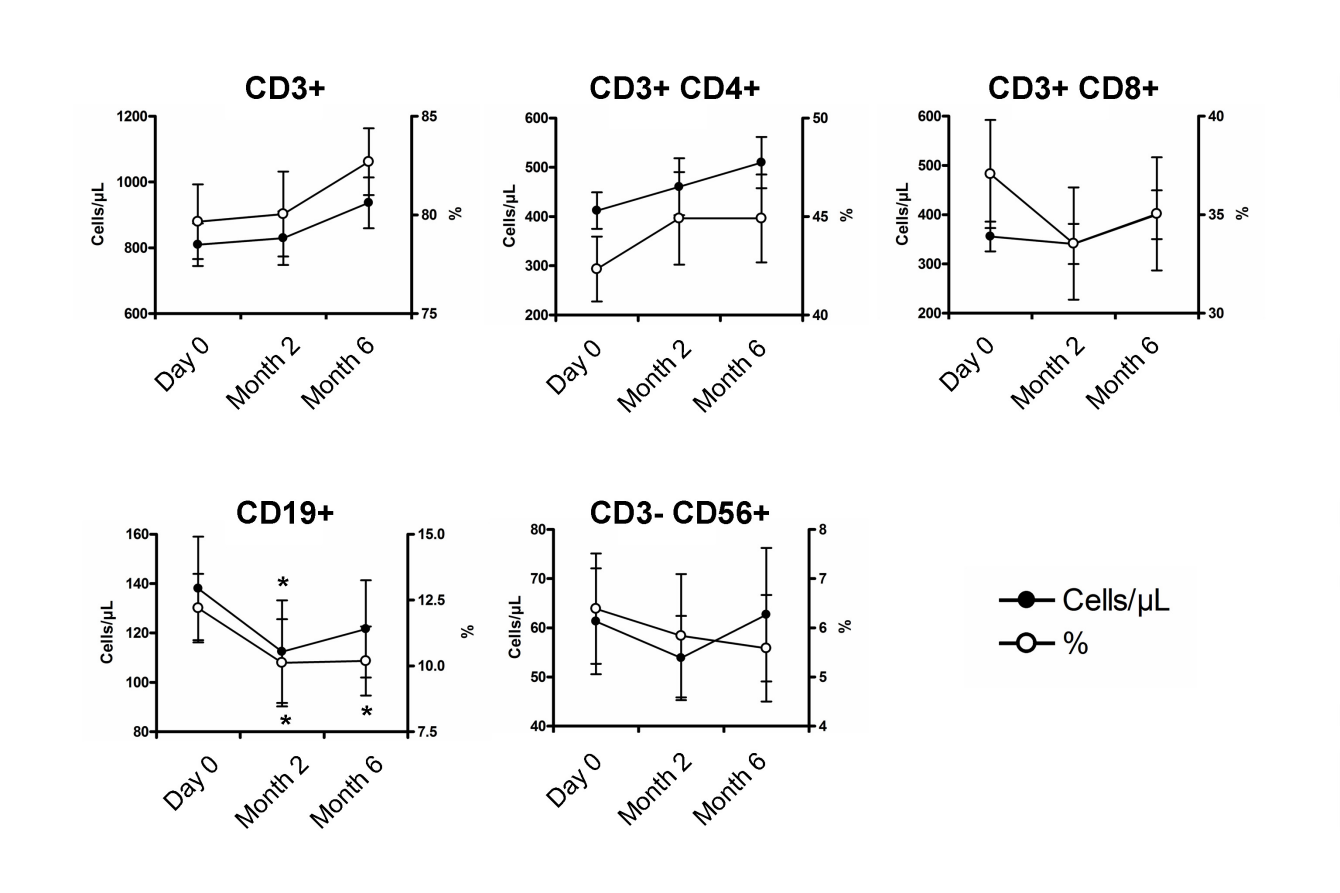
**Evolution of peripheral blood lymphocytes with vitamin D supplementation**. Time evolution of the proportion and the absolute number of CD3^+ ^T cells, CD4^+ ^and CD8^+ ^T cells, CD19^+ ^B cells and CD3^-^CD56^+ ^NK cells. At 2 and 6 months, the proportion and absolute number of CD3^+ ^T cells, CD4^+ ^and CD8^+ ^T cells and CD3^-^CD56^+ ^NK cells remained stable, while the CD19^+ ^B cell frequency and count significantly decreased at 2 months. Mean ± standard error of the mean (SEM) is shown. *P < 0.05 versus Day 0; Wilcoxon test.

The impact of vitamin D supplementation on the proportion and the absolute number of CD3^+ ^T cells, CD4^+ ^and CD8^+ ^T cells, CD19^+ ^B cells and CD3^-^CD56^+ ^NK cells is shown in Figure 2. The mean proportions and absolute numbers at baseline were 80 ± 8% and 810 ± 274/mm^3 ^for CD3^+ ^T cells, 42 ± 7% and 412 ± 157/mm^3 ^for CD4^+ ^T cells, 37 ± 12% and 356 ± 128/mm^3 ^for CD8^+ ^T cells, 12 ± 6% and 138 ± 89/mm^3 ^for CD19^+ ^B cells and 6 ± 5% and 61 ± 46/mm^3 ^for CD3^-^CD56^+ ^NK cells. At M2 and M6, the proportion and absolute number of CD3^+ ^T cells, CD4^+ ^and CD8^+ ^T cells and CD3^-^CD56^+ ^NK cells remained stable, while CD19^+ ^B cell frequency and counts significantly decreased at M2 (*P *< 0.05 for both) (Figure 2).

### Vitamin D supplementation induces a preferential increase of naïve CD4^+ ^T cells and a decrease of effector memory CD8^+ ^T cells and memory B cells

**Figure 3:**
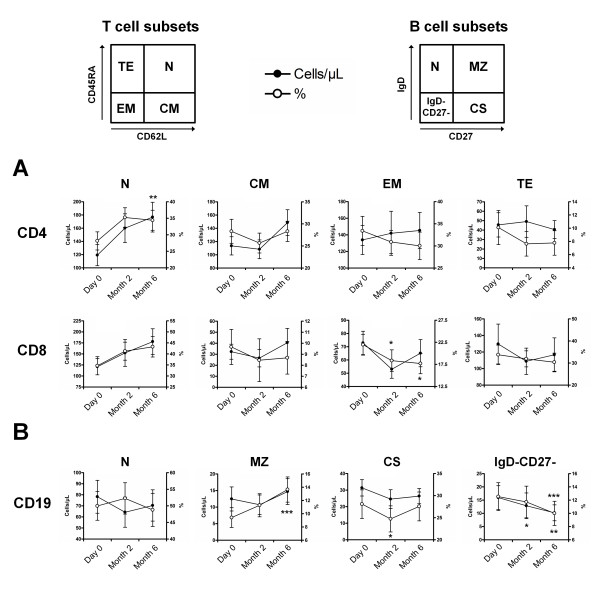
**Vitamin D supplementation induces a preferential increase of naïve CD4^+ ^T cells and a decrease of memory B cells**. Peripheral blood mononuclear cells **(**PBMCs) in the lymphocyte light-scatter gate were analyzed for T cell subsets using CD3, CD4, CD45RA and CD62L staining, and for B cell subsets using CD19, CD27 and IgD staining. (**A**) Time evolution of CD4^+ ^and CD8^+ ^T cell subsets. The T cell population includes naive (N; CD45RA+CD62L+), central memory (CM; CD45RA-CD62L+), effector memory and effector (EM; CD45RA-CD62L-), and terminally differentiated effector (TE; CD45RA+CD62L-). (**B**) Time evolution of CD19^+ ^B cell subsets. The B cell population includes naive (N; IgD+CD27-), marginal zone-like (MZ; IgD+CD27+), class-switched memory (CS IgD-CD27+), and IgD-CD27- B cells. Peripheral blood naïve CD4^+ ^T cells increased and IgD-CD27- memory B cells decreased under vitamin D supplementation. Mean ± standard error of the mean (SEM) is shown. *P < 0.05, **P < 0.01, ***P < 0.001 versus Day 0; Wilcoxon test.

Naïve CD4^+ ^T cells increased in frequency without reaching the significance level (*P *= 0.09 and *P *= 0.10, respectively) and in absolute number (*P *= 0.05 and *P *< 0.01, respectively) at M2 and M6 with vitamin D supplementation, while other CD4^+ ^T cell subsets remained stable. Effector memory CD8^+ ^T cells decreased in frequency (*P *= 0.02 and *P *= 0.01, respectively) but not in absolute number (*P *= 0.11 and *P *= 0.55, respectively) at M2 and M6 with vitamin D supplementation (Figure 3A).

Using IgD and CD27 surface markers, we also observed a decrease in IgD^-^CD27^+ ^class-switched memory B cell frequency (*P *< 0.05) and absolute number (*P *= 0.06) at M2 and in IgD^-^CD27^- ^memory B cell frequency (*P *= 0.11 and *P *< 0.001, respectively) and absolute number (*P *< 0.05 and *P *< 0.01, respectively) at M2 and M6, while IgD^+^CD27^+ ^marginal zone-like B cell frequency increased at M6 (*P *< 0.001) (Figure 3B).

### Vitamin D supplementation induces a significant increase of regulatory T cells

**Figure 4:**
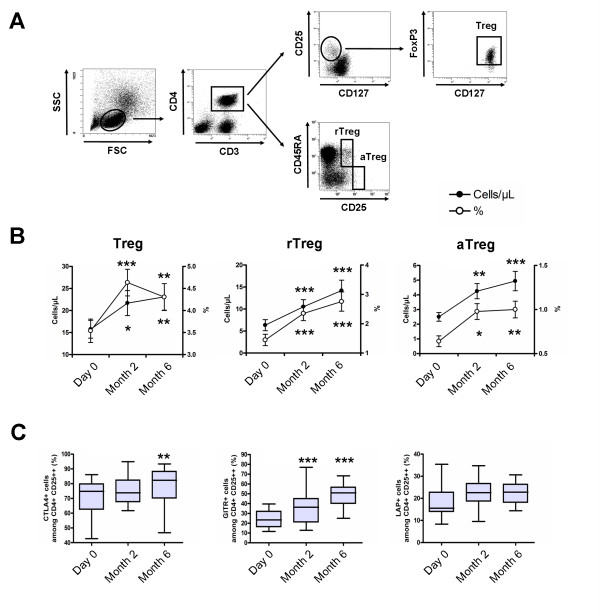
**Vitamin D supplementation induces a significant increase of regulatory T cells**. (**A**) Peripheral blood mononuclear cells **(**PBMCs) in the lymphocyte light-scatter gate were analyzed for CD3, CD4, CD25, CD127, CD45RA and FoxP3 staining. Regulatory T cells (Tregs), resting Tregs (rTregs) and activated memory Tregs (aTregs) were defined as CD3^+^CD4^+^CD25^hi^CD127^-^FoxP3^+ ^cells, CD3^+^CD4^+^CD25^++^CD45RA^+ ^cells and CD3^+^CD4^+^CD25^+++^CD45RA^- ^cells, respectively. (**B**) Time evolution of peripheral blood Tregs, rTregs and aTregs in percentage and absolute number. (**C**) Time evolution of CTLA4, GITR and LAP expression by Tregs. Peripheral blood Tregs, rTregs and aTregs increased under vitamin D supplementation, as did the expression of molecules associated with suppression of Tregs. Mean ± standard error of the mean (SEM) is shown in panel B. Box plots in panel C indicate median, interquartile ranges, minimum and maximum values. *P < 0.05, **P < 0.01, ***P < 0.001 versus Day 0; Wilcoxon test.

The impact of vitamin D supplementation on CD3^+^CD4^+^CD25^hi^CD127^-^FoxP3^+ ^Tregs and Treg subsets was evaluated (Figure 4A). The percentage and absolute count of Tregs at baseline was 3.5 ± 1.2% and 15 ± 8 cells/μL. The percentage of Tregs was increased at 4.6 ± 1.3% at M2 (*P *<0.001) and 4.3 ± 1.4% at M6 (*P *<0.01), and the absolute count of Tregs was increased at 23 ± 14 cells/μL (*P *<0.05) and 25 ± 14 cells/μL (*P *<0.01), respectively (Figure 4B). Analysis of CD45RA and CD25 expression on CD4^+ ^T cells revealed that this increase concerned both resting and activated memory Tregs (Figure 4B). The increase in Tregs was associated with an increased expression of molecules associated with Treg suppression (that is, CTLA4, GITR and LAP) (Figure 4C).

### Vitamin D supplementation induces a significant decrease of effector Th1 and Th17 cells

**Figure 5:**
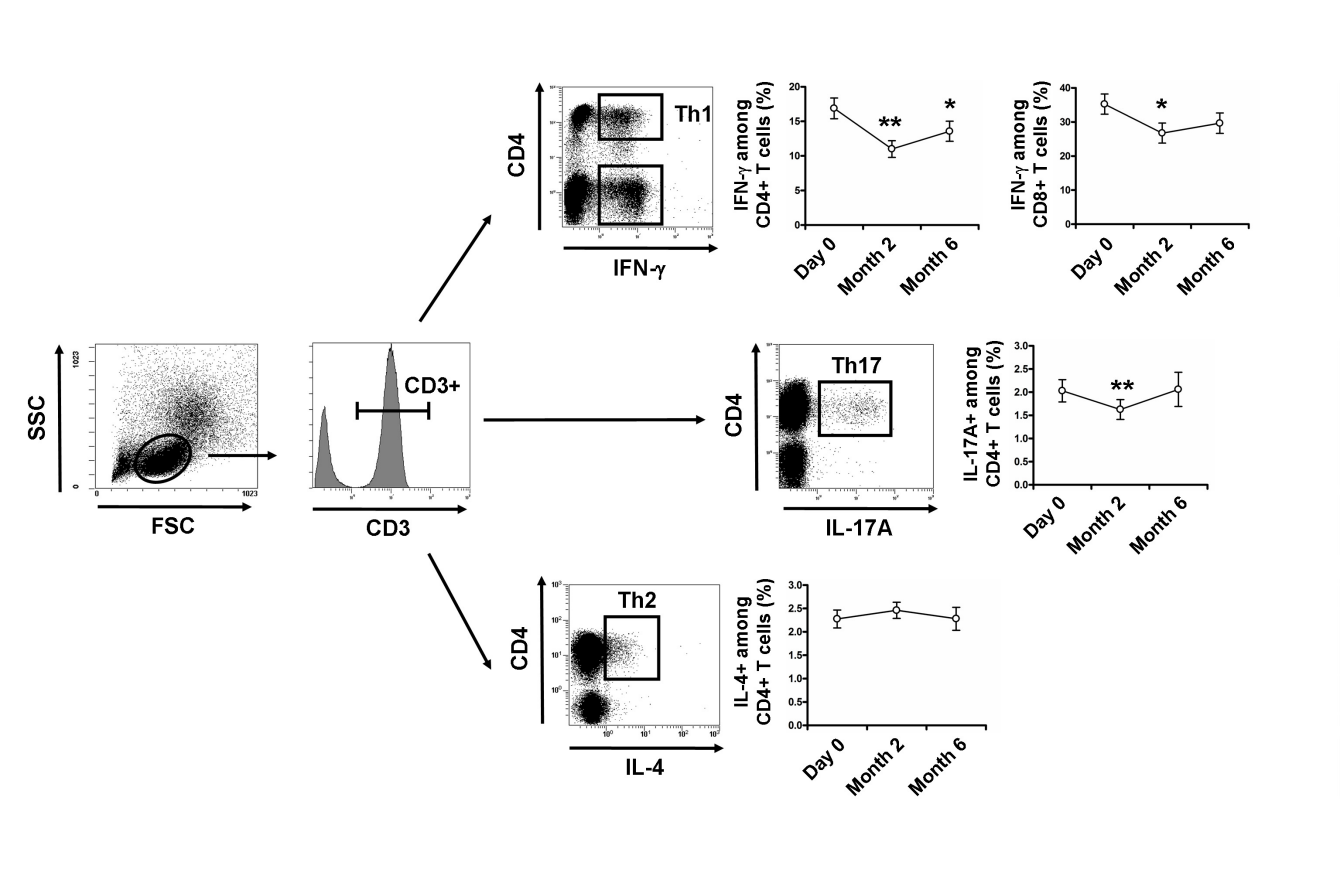
**Vitamin D supplementation induces a significant decrease in Th1 and Th17 cells**. Peripheral blood mononuclear cells **(**PBMCs) were stimulated for 4 hours with Phorbol myristate acetate (PMA) and ionomycin. After gating on CD3^+ ^T cells, frequencies of IFN-γ-producing CD4^+ ^(Th1) and CD8^+ ^T cells, IL-17A-producing CD4^+ ^T cells (Th17) and IL-4-producing CD4^+ ^T cells (Th2) were analyzed with vitamin D supplementation. Th1, IFN-γ-producing CD8^+ ^T cells and Th17 cells decreased after 2 months of vitamin D supplementation. Mean ± standard error of the mean (SEM) is shown. *P < 0.05, **P < 0.01, ***P < 0.001 versus Day 0; Wilcoxon test.

Decreases were observed in Th1 cells from 16.9 ± 6.7% at D0 to 11.0 ± 5.1% at M2, and to 13.6 ± 6.5% at M6 (*P *< 0.01 and *P *< 0.05, respectively), in IFN-producing CD8^+ ^T cells from 35.3 ± 13.2% at D0 to 26.8 ± 12.5% at M2, and to 29.7 ± 13.4% at M6 (*P *< 0.05 and *P *= 0.06, respectively), and in Th17 from 2.0 ± 1.1% at D0 to 1.6 ± 0.9% at M2 and to 2.0 ± 1.3% at M6 (*P *< 0.01 and *P *= 0.81, respectively) after vitamin D supplementation. The Th2 cells remained stable during follow-up (Figure 5).

## Discussion

No study to date has assessed the *in vivo *benefit of vitamin D supplementation in SLE patients. For the first time, we assessed the safety and the immunological effects of vitamin D supplementation in patients with SLE. We demonstrated that vitamin D was safe and induced a decrease of memory B cells, an increase of Tregs and a decrease of effector Th1 and Th17 cells.

First, we confirmed the high frequency of hypovitaminosis D in SLE patients. Recent studies have highlighted vitamin D insufficiency in SLE patients, with approximately 65% of the patients showing serum 25(OH)D levels < 30 ng/mL [19-21]. In the present study, we aimed to reach the recommended serum 25(OH)D ranges for bone metabolism, that is, 30 to 80 ng/mL, within the first 2 months. The schedule of vitamin D administration that we choose included a first phase of intensive supplementation for one month, followed by a maintenance phase. Regarding the evolution of serum levels of 25(OH)D, calcium and phosphorous and the occurrence of clinical events, this regimen was effective and safe. We included only SLE patients with low disease activity (SLEDAI ≤ 8) because we wanted to determine the specific effects of vitamin D in the absence of initiation or modification of associated therapy, such as an increase of the prednisone dosage or the use of immunosuppressive agents.

We demonstrated that vitamin D supplementation induced a beneficial effect *in vivo *on the perturbations of B cell and T cell homeostasis associated with SLE, by increasing Tregs and decreasing Th17 and Th1 cells, and memory B cells. Interestingly, the increase of Tregs concerned both resting Tregs and activated memory Tregs according to CD45RA and CD25 expression [22]. Our findings are supported by recently reported *in vitro *studies. 1,25(OH)_2 _vitamin D3, the active form of vitamin D, was shown to exert a marked inhibitory effect on adaptive immune cells, by inhibiting the T cell proliferation [23,24], the expression of IL-2 [24,25] and IFN-γ mRNA and protein in T cells [9,26,27], while promoting Th2-cell responses *in vitro *[28]. In our *in vivo *study, we did not observe a significant increase of Th2 cells under vitamin D supplementation, since this T cell subset remains stable during follow-up. 1,25(OH)_2 _vitamin D3 was also shown to inhibit Th17 responses, probably owing to its capacity to inhibit IL-23 production [9,29], and to induce the differentiation and/or expansion of FoxP3^+ ^Tregs and an increased expression of CTLA4 [9,30-32]. Finally, we observed a decrease of IgD^-^CD27^+ ^and IgD^-^CD27^- ^memory B cells and a decrease of anti-dsDNA autoantibody levels in vitamin D-treated patients. Consistent with these findings, 1,25(OH)_2 _vitamin D3 was shown to decrease B cell proliferation, plasma-cell differentiation and IgG secretion *in vitro *[12,23]. The mechanisms by which 1,25(OH)_2 _vitamin D3 exerts its immunomodulatory effect on B cells remains unclear.

Although too preliminary to be presented in the present study, we have performed a transcriptome analysis of PBMCs at D0 versus M2 after vitamin D supplementation on ten randomly selected patients, in order to provide additional insights into the immunological effects of vitamin D in SLE (Additional file 1). Using independent component analysis (ICA) [33] and gene set enrichment analysis (GSEA) dataset [34,35], we identified 48 molecular signatures that were differentially expressed between D0 and M2, with 34 up- and 14 down-regulated signatures (see Figure S1 inAdditional file 1. This preliminary finding suggests an effect of vitamin D supplementation on the immune system. Among the identified signatures, we observed the down-regulation of RNA polymerase functions and histone expression and the up-regulation of the TP53/CDKN1A-related pathway that represent interesting pathways to explore in the future, owing to their possible involvement with a decrease in the accumulation of autoantigens and the activation and proliferation of autoreactive T and B lymphocytes. Also, the up-regulation of the TP53/CDKN1A-related pathway is interesting because CDKN1A is a potent cyclin-dependent kinase inhibitor that functions as a regulator of cell cycle progression [36].

## Conclusions

Vitamin D supplementation provides beneficial immunological effects in patients with SLE, with a decrease of memory B cells and effector T cells and an increase of regulatory T cells. Our results must be interpreted with caution in the absence of well-designed randomized controlled trials, particularly for a relevant clinical endpoint. In addition, we focused on the immunological effects of vitamin D after two months and six months of supplementation, but earlier time points should bring additional information, particularly for gene expression analysis, calling for further studies.

### Significance and innovation

Vitamin D supplementation using 100,000 IU of cholecalciferol per week for 4 weeks, followed by 100,000 IU of cholecalciferol per month for 6 months, was well tolerated.

Vitamin D induced a preferential increase of naïve CD4^+ ^T cells, an increase of regulatory T cells, a decrease of effector Th1 and Th17 cells, and a decrease of memory B cells and anti-DNA antibodies.

This preliminary study suggests a beneficial role of vitamin D in SLE patients and needs to be confirmed in randomized controlled trials.

## Abbreviations

APC: allophycocyanin; CDKN1A: cyclin-dependent kinase inhibitor 1A; ECD: Phycoerythrin-Texas Red; ELISA: enzyme-linked immunosorbent assay; FITC: fluorescein isothiocyanate; FoxP3: forkhead box P3; GSEA: gene set enrichment analysis; ICA: independent component analysis; IFN: interferon; IL: interleukin; 25(OH)D: 25-hydroxyvitamin D; PBMC: peripheral blood mononuclear cell; PCy7: Phycoerythrin-Cyanin 7; PE: Phycoerythrin; PMA: phorbol myristate acetate; SELENA-SLEDAI: Safety of Estrogens in Lupus Erythematosus National Assessment-Systemic Lupus Erythematosus Disease Activity Index; SLE: systemic lupus erythematosus; Th17: T helper lymphocytes producing IL-17; Tregs: regulatory T cells.

## Competing interests

The authors declare that they have no competing interests.

## Authors' contributions

BT, ND, YS, GG, PC and NCC devised and performed experiments; BT, PC and NCC provided patient referrals; BT, ND, YS, WC, GG, NZ, KM, MR, WC, LM, JCP, AS, DK, DS, PC and NCC interpreted results; and BT, PC and NCC designed the research and wrote the paper. All authors have read and approved the manuscript for publication.

## Supplementary Material

Additional file 1 **Microarray gene expression profile analysis**. This file contains additional material and a methods section that describes microarray gene expression profile analysis, and Figure S1 that illustrates clustering analysis of significant modulated signatures in patients at month 2 compared to baseline. Click here for file
