# Supplementary material for: Restoration of regulatory and effector T cell balance and B cell homeostasis in systemic lupus erythematosus patients through vitamin D supplementation
Source: Arthritis Res Ther. 2012 Oct 17;14(5):R221. doi: 10.1186/ar4060 (PMC3580532; doi:10.1186/ar4060)

**Additional Material and Methods**

***Microarray gene expression profile analysis***

RNAs were generated from PBMCs of 12 SLE patients randomly selected among the 20 patients, at baseline and after 2 months of vitamin D supplementation, using the [NucleoSpin RNA II](http://www.mn-net.com/tabid/1324/default.aspx) kit (Macherey-Nagel, Hoerd, France) according to the manufacturer’s instructions. RNA integrity was assessed using an Agilent Bioanalyser showing a quality RNA integrity number of 8–10 (Agilent Technologies). RNA yield was assessed using a NanoDrop 1000 spectrophotometer (NanoDrop Products, Thermo Fisher Scientific). Each patient included in the gene expression profile analysis was selected through a randomization process using the R.2.10.1 statistical package (R Development Core Team, R: A Language and Environment for Statistical Computing, 2009, Vienna, Austria, http://www.R-project.org).

Total RNA was amplified and converted to biotinylated cRNA according to the manufacturer’s protocol (Illumina TotalPrep RNA Amplification Kit; Ambion). Labelled cRNA were hybridized overnight to Illumina Human HT-12 V3 BeadChip arrays (Illumina), which contained more than 48,000 probes. The arrays were then washed, blocked, stained and scanned on an Illumina BeadStation following the manufacturer’s protocols. Illumina BeadStudio software (Illumina) was used to generate signal intensity values from the scans.

Two patients with modulation profiles that were too divergent from the others were excluded from the clustering analysis. Data were normalized according to the quantile method on 6032 selected transcripts that were significantly detected (p<0.05) in at least one condition in 60% of patients.

Potential molecular signatures were extracted using Independent Component Analysis (ICA). Those signatures were added to the MSigDB C2 and C5 signature databases and tested for their significance on our microarray data using Gene Set Enrichment Analysis (GSEA) software. Statistically significant molecular signatures [false discovery rate (FDR) q.value<0.05] were annotated for their Gene Ontology (GO) terms and Kyoto Encyclopedia of Genes and Genomes (KEGG) pathways enrichment.

Graphical clustering was performed using distance calculus based on Jaccard index, clusters were controled and validated using dunn and connectivity measures.

**Additional Figure S1.** **Clustering analysis of significant modulated-signatures in patients at month 2 compared to baseline.** Using an ICA algorithm and GSEA dataset, we identified 48 signatures that were differentially regulated between day and month 2, including 34 up- and 14 down-regulated signatures (FDR<0.05). Cluster Analysis based on Jaccard index and signature annotations reveals groups of signatures sharing biological processes as shown on this graph. Twenty-four signatures are involved in the immune response (21 up and three down-regulated), and seven up-regulated signatures in energetic processes. In addition, a cluster of five down-regulated signatures is associated with RNA polymerase functions and nucleosome formation, whereas a CDKN1A/TP53-related signature was up-regulated.


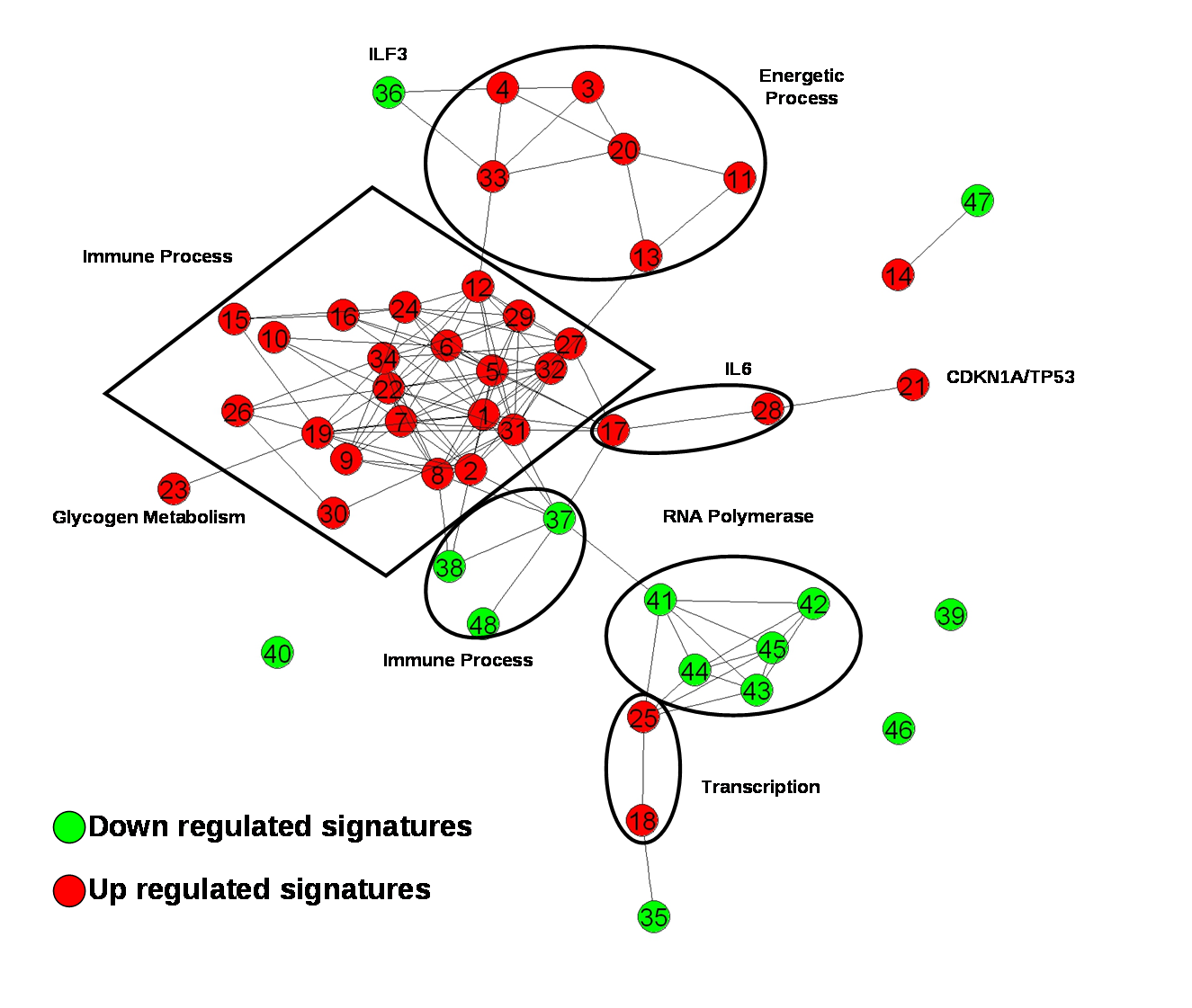

Supplement: Additional file 1 — Microarray gene expression profile analysis. This file contains additional material and a methods section that describes microarray gene expression profile analysis, and Figure S1 that illustrates clustering analysis of significant modulated signatures in patients at month 2 compared to baseline. [file ar4060-S1.DOC]
